# Excitatory and inhibitory projections in parallel pathways from the inferior colliculus to the auditory thalamus

**DOI:** 10.3389/fnana.2014.00124

**Published:** 2014-11-05

**Authors:** Jeffrey G. Mellott, Nichole L. Foster, Andrew P. Ohl, Brett R. Schofield

**Affiliations:** ^1^Department of Anatomy and Neurobiology, Northeast Ohio Medical UniversityRootstown, OH, USA; ^2^School of Biomedical Sciences, Kent State UniversityKent, OH, USA

**Keywords:** tectothalamic, medial geniculate, GABA, GAD, lemniscal, non-lemniscal, auditory system

## Abstract

Individual subdivisions of the medial geniculate body (MG) receive a majority of their ascending inputs from 1 or 2 subdivisions of the inferior colliculus (IC). This establishes parallel pathways that provide a model for understanding auditory projections from the IC through the MG and on to auditory cortex. A striking discovery about the tectothalamic circuit was identification of a substantial GABAergic component. Whether GABAergic projections match the parallel pathway organization has not been examined. We asked whether the parallel pathway concept is reflected in guinea pig tectothalamic pathways and to what degree GABAergic cells contribute to each pathway. We deposited retrograde tracers into individual MG subdivisions (ventral, MGv; medial, MGm; dorsal, MGd; suprageniculate, MGsg) to label tectothalamic cells and used immunochemistry to identify GABAergic cells. The MGv receives most of its IC input (~75%) from the IC central nucleus (ICc); MGd and MGsg receive most of their input (~70%) from IC dorsal cortex (ICd); and MGm receives substantial input from both ICc (~40%) and IC lateral cortex (~40%). Each MG subdivision receives additional input (up to 32%) from non-dominant IC subdivisions, suggesting cross-talk between the pathways. The proportion of GABAergic cells in each pathway depended on the MG subdivision. GABAergic cells formed ~20% of IC inputs to MGv or MGm, ~11% of inputs to MGd, and 4% of inputs to MGsg. Thus, non-GABAergic (i.e., glutamatergic) cells are most numerous in each pathway with GABAergic cells contributing to different extents. Despite smaller numbers of GABAergic cells, their distributions across IC subdivisions mimicked the parallel pathways. Projections outside the dominant pathways suggest opportunities for excitatory and inhibitory crosstalk. The results demonstrate parallel tectothalamic pathways in guinea pigs and suggest numerous opportunities for excitatory and inhibitory interactions within and between pathways.

## Introduction

The projections from the inferior colliculus (IC) to the medial geniculate body (MG) have been described as 3 parallel pathways: (1) a “lemniscal” or “tonotopic” pathway; (2) a “polysensory” pathway; and (3) a “diffuse” pathway (Calford and Aitkin, 1983; Rouiller, 1997). The pathways reflect subdivision-specific connections from the IC to the MG and from the MG to auditory cortex (and other forebrain targets) and are considered to serve different functions in hearing (Oliver and Hall, 1978a,b; Calford and Aitkin, 1983; Redies et al., 1989; Redies and Brandner, 1991; Hu et al., 1994; de Ribaupierre, 1997; Bartlett and Smith, 1999, 2002; Edeline et al., 1999; He, 2001; Hu, 2003; Smith et al., 2007; Anderson et al., 2009; Lee and Sherman, 2010; Anderson and Linden, 2011; Edeline, 2011; Venkataraman and Bartlett, 2013). The lemniscal pathway has been associated with primary-like representation of sound. It is formed primarily by projections from central IC (ICc) to ventral MG (MGv) and from there to tonotopically organized parts of the auditory cortex (de Ribaupierre, 1997). The diffuse pathway has been associated with complex sounds and detecting change in context-dependent signals (de Ribaupierre, 1997). The diffuse pathway involves projections from IC dorsal cortex (ICd) to dorsal MG (MGd) and from there to non-tonotopic secondary and temporal auditory cortical areas (de Ribaupierre, 1997). Finally, the polysensory pathway has been associated with multimodal processing, reflecting inputs from auditory as well as other sensory systems (Love and Scott, 1969). The polysensory pathway is unique among the three pathways in several ways. While it is closely associated with a single MG subdivision (the medial MG, MGm), it receives substantial inputs from 2 IC subdivisions (the ICc and the IC lateral cortex, IClc). The polysensory pathway also differs from the other pathways in having much broader projections to the forebrain, terminating widely across all areas of auditory cortex. Moreover, these thalamocortical projections terminate most heavily in cortical layer I, whereas the thalamocortical projections in the lemniscal and diffuse pathways terminate most heavily in the middle cortical layers (III–IV). While tectothalamic projections show some overlap (e.g., the ICc contributes to both the lemniscal and polysensory pathways), the general segregation is assumed to underlie substantial physiological and functional differences between these pathways.

One of the most striking discoveries about the tectothalamic pathways has been the detection of an inhibitory component arising from GABAergic IC cells (Winer et al., 1996; Peruzzi et al., 1997; Bartlett and Smith, 1999; Smith et al., 2007; Mellott et al., 2014). GABAergic tectothalamic cells are found throughout the IC and, depending on the species, constitute 20–50% of the tectothalamic cells (cats: 20%, Winer et al., 1996; rats: 40%, Peruzzi et al., 1997; guinea pigs: 22%; Mellott et al., 2014). The remaining tectothalamic cells are glutamatergic, providing ascending excitation to the MG. Physiological studies have shown that the ascending excitatory and inhibitory inputs are integrated in different ways by neurons in different MG subdivisions, supporting the proposed functional distinctions between the MG subdivisions and the associated parallel pathways (Smith et al., 2007). However, previous anatomical studies of the GABAergic projections were based on tracer injections that included two or more subdivisions of the MG and thus did not address whether the GABAergic projections targeted a specific subdivision in the MG (Winer et al., 1996; Peruzzi et al., 1997; Mellott et al., 2014). An understanding of the various functions of the MG subdivisions and their ascending projections will require a clear delineation of both the excitatory and inhibitory projections they receive from the IC.

In the present study, we combine retrograde transport from individual MG subdivisions with immunochemistry to distinguish GABAergic from non-GABAergic tectothalamic cells. We completed the studies in guinea pigs, which have recently been subjects of both anatomical and physiological studies of the MG subdivisions but which have not been examined with respect to the parallel tectothalamic pathways (Anderson et al., 2006, 2007). The more recent study distinguished a “suprageniculate” subdivision (MGsg), that has been described in some other species but is often included with the MGd or the MGm. Support for distinguishing this subdivision in the context of tectothalamic projections comes from preliminary studies suggesting that the MGsg differs from the other subdivisions (MGv, MGd and MGm) in receiving very little GABAergic input from the IC (Mellott and Schofield, 2011). Our findings suggest that the IC projections to individual MG subdivisions in guinea pigs are similar to those described in other species. In addition, GABAergic cells contribute to each of these pathways. In general, both the GABAergic (presumed inhibitory) and the non-GABAergic (presumed excitatory) projections from a particular IC subdivision have strong projections to specific MG subdivisions and smaller projections to other regions of the MG. These latter projections could provide for both excitatory and inhibitory cross-talk between the parallel pathways.

## Materials and methods

All procedures were conducted in accordance with the Northeast Ohio Medical University Institutional Animal Care and Use Committee and NIH guidelines. Results are described from ten adult pigmented guinea pigs (Elm Hill Labs; Chelmsford, MA, USA) of either gender weighing 317–1000 g (most animals were age 5 weeks to 4 months; 1 animal was ~2 years old). Efforts were made to minimize the number of animals and their suffering.

### Surgery

Each animal was anesthetized with isoflurane (4–5% for induction, 1.75–3% for maintenance) in oxygen. The head was shaved and disinfected with Betadine (Purdue Products L.P., Stamford, CT, USA). Atropine sulfate (0.08 mg/kg i.m.) was given to minimize respiratory secretions and Ketofen (ketoprofen, 3 mg/kg i.m.; Henry Schein, Melville, NY 11747, USA) was given for post-operative pain management. Moisture Eyes PM ophthalmic ointment (Bausch, Lomb, Rochester, NY, USA) was applied to each eye to protect the cornea. The animal’s head was positioned in a stereotaxic frame. Body temperature was maintained with a feedback-controlled heating pad. Sterile instruments and aseptic techniques were used for all surgical procedures. An incision was made in the scalp and the surrounding skin was injected with Marcaine (0.25% bupivacaine with epinephrine 1:200,000; Hospira, Inc., Lake Forest, IL, USA), a long-lasting local anesthetic. A craniotomy was made over the desired target coordinates using a dental drill. Following the tracer injection, Gelfoam (Harvard Apparatus, Holliston, MA, USA) was placed in the craniotomy site and the scalp was sutured. The animal was then removed from the stereotaxic frame and placed in a clean cage. The animal was monitored until it could walk, eat and drink without difficulty.

### Retrograde tracers

Fluorescent tracers (red fluorescent RetroBeads [“red beads”] and green fluorescent RetroBeads [“green beads”], Luma-Fluor, Inc., Naples, FL, USA; FluoroGold, FluoroChrome, Inc., Englewood, CO, USA) were deposited into the MG via stereotaxic coordinates. For most experiments, a Hamilton microsyringe (1 µl; Hamilton, Reno, NV, USA) or a micropipette (tip diameter 25–35 μm) attached to a Nanoliter Injector (World Precision Instruments, Sarasota, FL, USA) was used to deposit one of the tracers into the MG (Table 1). Each syringe was dedicated to a single tracer. Injections were small in volume, < 70 nl, to better ensure the deposit was contained primarily or exclusively within one subdivision of the MG. In order to limit the spread of tracer into neighboring nuclei, the volume injected at each site was designed to account for the diffusibility of each tracer (Schofield, 2008). In one animal, FluoroGold was deposited by iontophoresis through a micropipette (tip diameter 20 μm, +1.5 μA current, 15 min, 50% duty cycle) (Table 1).

**Table 1:** **Summary of the tracers, volumes injected, and spread of injection sites into MG subdivisions after injections into left (L) and/or right (R) MG**.

				Extent of injection site			
Case	Side	Tracer	Total volume	MGv	MGd	MGm	MGsg
GP689	L	RB	69 nl	-	-	X	(x)
GP689*	R	GB	69 nl	-	-	X	-
GP693*	L	RB	46 nl	-	X	-	-
GP695*	L	RB	27.6 nl	X	-	-	-
GP696*	L	RB	27.6 nl	-	-	-	X
GP698*	L	RB	18.4 nl	-	-	-	X
GP702	R	FG	ion^#^	X	(x)	-	-
GP712	L	RB	50 nl	-	X	-	(x)
GP718*	L	FG	50 nl	-	X	-	-
GP719*	L	FG	50 nl	X	-	-	-
GP723	L	FB	50 nl	(x)	X	-	-

X = significant involvement of the deposit. (x) = indicates minor involvement of the listed MG subdivision. – = no involvement of the listed MG subdivision. ion^#^ = iontophoretic injection. nl = nanoliter. * indicates cases used for quantitative analysis.

### Perfusion and tissue processing

Five to thirteen days after surgery, the animal was deeply anesthetized with isoflurane and perfused transcardially with Tyrode’s solution, followed by 250 ml of 4% paraformaldehyde in 0.1 M phosphate buffer, pH 7.4 and then by 250 ml of the same fixative with 10% sucrose. The brain was removed and stored at 4°C in fixative with 25–30% sucrose for cryoprotection. The following day the brain was prepared for processing by removing the cerebellum and blocking the remaining piece with transverse cuts posterior to the superior olive and anterior to the auditory cortex. Each piece of tissue was frozen and cut on a sliding microtome into 40 or 50 µm thick transverse sections that were collected serially in six sets.

Putative GABAergic cells were stained with immunochemistry for glutamic acid decarboxylase (GAD; Nakamoto et al., 2013). Briefly, the sections were pretreated with normal goat serum to limit non-specific labeling, then exposed (1–2 days at 4°C) to mouse anti-GAD monoclonal antibody (GAD67; #MAB5406 Millipore, diluted 1:1000 to 1:100). The sections were treated with 1% biotinylated goat anti-mouse antibody (Vector Laboratories, Burlingame, CA, USA: BA-9200) and labeled with streptavidin conjugated to a fluorescent marker (AlexaFluor 488 [green] or AlexaFluor 647 [near-infrared], Invitrogen, Carlsbad, CA, USA). For transversely cut cases, a series of sections adjacent to the one used for tracer analysis was stained to facilitate identification of IC and MG subdivisions. The IC and the MG do no coexist in the transverse plane so sections with IC tissue could be separated from sections with MG tissue. The method of Coote and Rees (2008) was used to stain IC sections with antibodies to brain nitric oxide synthase (bNOS) and then identify IC subdivisions. The method of Anderson et al. (2007) was used on MG sections to reveal cytochrome oxidase activity and to identify subdivisions of the MG. In one case (GP723) the tissue was cut in the sagittal plane. Because the IC and the MG coexist in the sagittal plane, one series was stained with bNOS to identify the IC subdivisions and the other series was stained with cytochrome oxidase to identify the MG subdivisions. These series were on either side of the tracer-analyzed series. Stained sections were mounted on gelatin-coated slides, allowed to dry and coverslipped with DPX (Sigma).

### Data analysis

Subdivisions of the MG were identified by their patterns of staining with cytochrome oxidase (Anderson et al., 2007). IC subdivisions were identified by the differential expression of bNOS, as detailed in Coote and Rees (2008). The borders of the ICc were clarified by observation at high power to identify disc-shaped cells that stain for bNOS and that are characteristic of the ICc (Coote and Rees, 2008). Immunostaining revealed GAD-immunoreactive (GAD+) cells and boutons throughout the IC. Immunopositive cells were labeled intensely and were readily distinguished from immunonegative cells. The GAD immunostain was also readily visible in tracer-labeled cells, making it straightforward to distinguish GAD+ vs. GAD-negative staining in the retrogradely-labeled cells, including cells that contained two different retrograde tracers.

The location and extent of each injection site was determined by comparison of the tracer deposit with borders of MG subdivisions identified in sections stained for cytochrome oxidase (Anderson et al., 2007). Results from seven injections (4 RB; 1 GB; 2 FG) that also had robust immunostaining were used for quantitative analysis (Table 1). Labeled cells in the IC were plotted with a Neurolucida reconstruction system (MBF Bioscience, Williston, VT, USA) attached to a Zeiss Axioplan II microscope (Carl Zeiss MicroImaging, Inc., Thornwood, NY, USA) or a Zeiss AxioImager Z2 with an attached Apotome II (Zeiss). For each case, every labeled cell was plotted in the ipsilateral IC across a series of transverse sections (every sixth section). Each combination of tracer and immunolabel was plotted with a unique marker. The results of these plots were used for a quantitative summary of the distributions of the labeled cells.

In some cases, the anti-GAD staining did not fully penetrate the tissue, resulting in a central layer in the section where GAD staining was absent. Sections cut at 40–50 µm thickness typically shrink to 20–30 µm thickness due to tissue processing and dehydration prior to mounting on slides. In some of our cases, the GAD staining was robust only 5–10 µm from each surface, leaving an unstained or poorly stained central layer typically 10–15 µm thick. Data from these cases were plotted with the Neurolucida system and a 63X objective (NA = 1.4), with special attention to focusing on the center of the soma when plotting the symbol for a particular cell. This approach provides sufficient resolution in the z plane (section depth) to allow subsequent filtering of the data by depth. After the data were plotted, the X, Y, and Z coordinates of all markers from each subdivision of each tissue section were exported from Neurolucida to Microsoft Excel and sorted based on the Z coordinate. The depth of penetration of the GAD labeling was assessed under the 63X objective for each subdivision of each section to determine the range of depths (measured from the top surface of the section) where GAD staining was robust. This yielded 2 zones of data from each section (1 associated with each surface), and a central zone that was not stained with GAD. All markers in the central, unstained zone were excluded from further analyses.

Figures showing the distribution of labeled cells were created with Neurolucida software (MBF Bioscience) and refined with Adobe Illustrator (Adobe Systems, Inc., San Jose, CA, USA). Photomicrographs were captured using either a Zeiss AxioImager Z1 fluorescence microscope and AxioCam HRm or HRc cameras (Zeiss) or a Zeiss Axioskop fluorescence microscope and Magnafire camera (Optronics, Goleta, CA, USA). Adobe Photoshop (Adobe Systems) was used to add scale bars, crop images, erase background around tissue sections, adjust intensity levels and colorize monochrome images.

## Results

We combined retrograde tracing and immunolabeling for GAD to identify GABAergic IC cells that project to individual subdivisions of the MG. While our main objective was to distinguish the GABAergic vs. non-GABAergic components of the tectothalamic pathways, it was necessary to first establish the overall patterns of connections between the IC subdivisions and the MG subdivisions. As described in the Introduction, a suprageniculate subdivision has been distinguished in several species, including guinea pigs. We continue this distinction and, for the sake of discussion, group the MGsg with the MGd as part of the diffuse pathway. We first describe the injection sites and evidence for parallel pathways without regard to GAD-immunoreactivity. We then describe the same experimental cases with attention to the presence or absence of GAD immunostaining in the retrogradely labeled cells.

### Injection sites and evidence for parallel tectothalamic pathways

**Figure 1:**
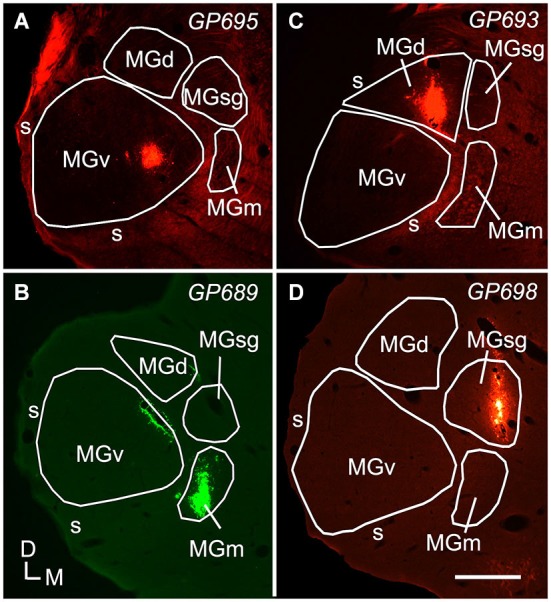
**Photomicrographs showing representative deposits of Red Beads (RB) or Green Beads (GB) in four subdivisions of the medial geniculate body (MG)**. **(A)** A deposit of RB contained within the left MGv. The bright fluorescence on the dorsolateral edge of the section is imaging artifact resulting from tissue damage during sectioning; the tracer deposit is the bright spot within the MGv. **(B)** A deposit of GB contained within the right MGm. Additional green fluorescence is seen around the margins of a blood vessel along the dorsomedial border of the ventral MG (v); this represents spread of beads that does not result in retrogradely labeled cells. The tracer was deposited in the right MG; the image is reversed left to right to facilitate comparisons with the other panels. **(C)** A deposit of RB contained within the MGd. **(D)** A deposit of RB contained within the left MGsg. Experiment numbers (e.g., GP695) are shown in each panel (cf. Table 1). Scale bar = 0.5 mm. D—dorsal; MGd—dorsal division of the MG; M—medial; MGm—medial division of the MG; s—shell of the MG; L—lateral; SC—superior colliculus; MGsg—suprageniculate division of the MG; MGv—ventral division of the MG.

The results are based on tracer deposits in 11 MGs (Table 1). Most of the injections were isolated to one subdivision of the MG (Figure 1). Quantitative data were derived from 7 cases. Four cases had deposits that were centered in a particular MG subdivision but spread slightly into an adjacent subdivision. The number of cells labeled by the encroachment was probably very small, but because the exact number could not be determined, these cases were excluded from quantitative analysis. Nonetheless, the overall labeling patterns in these cases were very similar to those with more restricted injections, and thus serve to confirm the findings. The number of labeled cells varied between cases, but the overall patterns and percentages of cells in IC subdivisions were consistent between animals and between different tracers for injections in a given subdivision.

**Figure 2:**
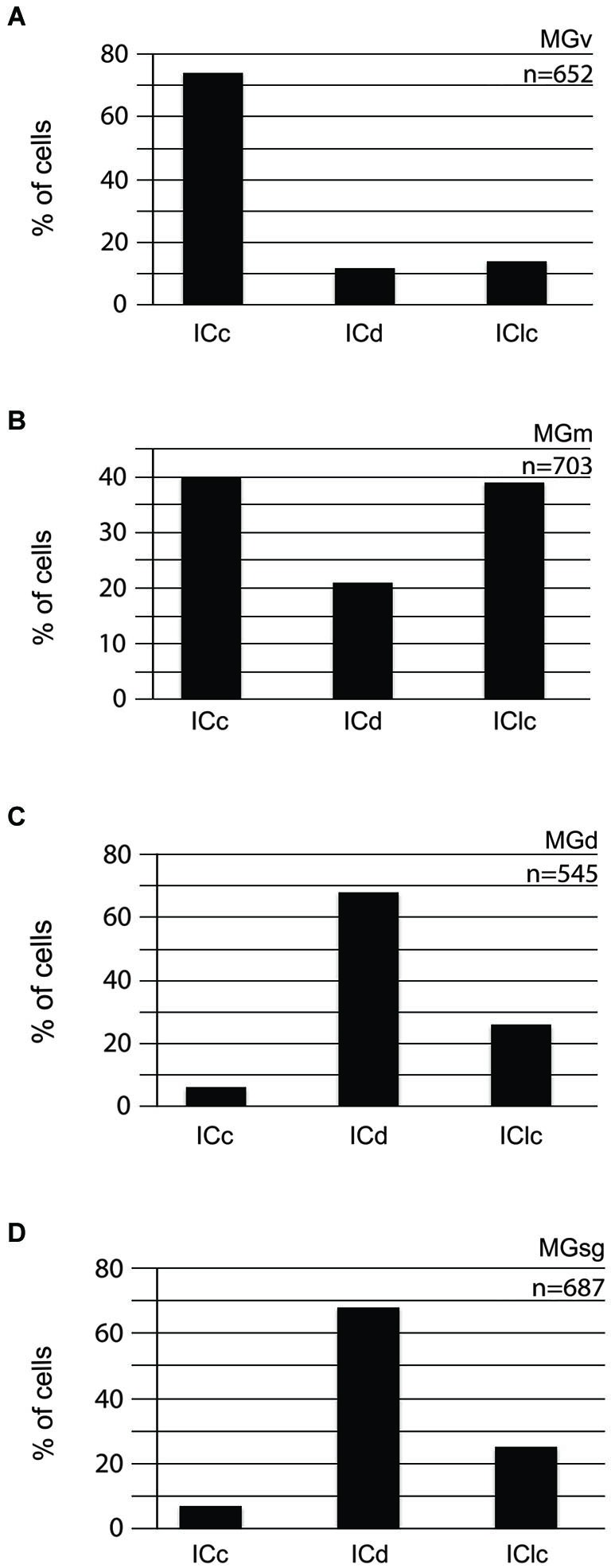
**Histograms showing the distribution of cells in the various subdivisions of the IC that project to the MGv (*n* = 2) (A), MGm (*n* = 1) (B), MGd (*n* = 2) (C), or MGsg (*n* = 2) (D)**. The *y* axis reflects the proportion of labeled cells in each IC subdivision as a percentage of all the labeled cells in the IC. *n* = the # of IC cells counted for injections into the indicated MG subdivision. ICc—IC central nucleus of the IC; ICd—IC dorsal cortex; IClc—IC lateral cortex; MGd—dorsal division of the MG; MGm—medial division of the MG; MGsg—suprageniculate division of the MG; MGv—ventral division of the MG.

Injections into different MG subdivisions yielded distinct distributions of labeled cells in the IC, supporting the idea of parallel but different pathways to each MG subdivision (Figure 2). Tracer injections into the MGv labeled cells across the IC, with a majority (three-fourths) located in the ICc (Figure 2A). A very different pattern followed injections into the MGm, where the majority of labeled cells were split nearly equally between two IC subdivisions (40% in the ICc and 39% in the IClc (Figure 2B). Injections into the MGd or the MGsg produced very similar results, with a majority of labeled cells located in the ICd (Figures 2C,D). Also in both situations, there were very few labeled cells in the ICc (Figures 2C,D). The similarities in these distributions (and their distinct difference from results of injections into the other 2 MG subdivisions) provides the rationale for grouping the MGd and the MGsg results together (associated with the “diffuse” pathway as described in the Introduction). The results of GAD immunochemistry, described below, will provide the basis for distinguishing the MGd from the MGsg.

### GAD-positive (GAD+) and GAD-negative tectothalamic cells

**Figure 3:**
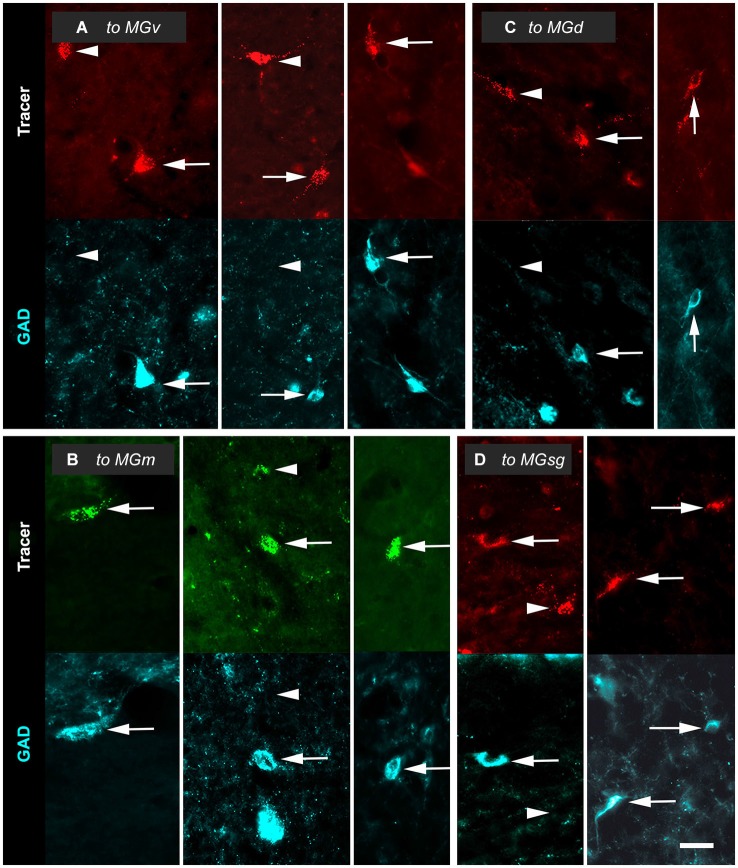
**Paired photomicrographs showing retrogradely-labeled cells in the inferior colliculus (IC) that are GAD-immunopositive (GAD+; arrows) or GAD-negative (arrowheads)**. The top row in each pair shows cells retrogradely labeled by either Red Beads or Green Beads. The bottom row in each pair shows the same field viewed for immunoreactivity to GAD (cyan). **(A)** Cells that project to the ipsilateral ventral division of the medial geniculate body (MGv). Images are taken from the IC central nucleus (ICc, left column); the IC dorsal cortex (ICd; middle column) and the IC lateral cortex (IClc; right column). GP695. **(B)** Cells that project to the ipsilateral medial division of the medial geniculate body (MGm). Images in the left, middle and right columns are from the ICc, ICd and IClc, respectively. GP689. **(C)** Cells that project to the ipsilateral dorsal division of the medial geniculate body (MGd). Images are from the ICd (left column) and IClc (right column). GP693. **(D)** Cells that project to the ipsilateral suprageniculate division of the medial geniculate body (MGsg). Images are from the ICd (left column) and IClc (right column). GP698. Scale bar = 50 µm.

Tracer injections into any of the MG subdivisions labeled GAD+ and GAD-negative IC cells. The GAD+ cells (Figure 3, arrows) were interpreted as GABAergic cells that project to the injection site. GAD-negative retrogradely-labeled cells (Figure 3, arrowheads) were often in close proximity to GAD+ cells. As described in Methods, our quantitative analyses included only those retrogradely-labeled cells at tissue depths that were successfully stained with anti-GAD immunostaining. Consequently, we interpreted these immunonegative cells as non-GABAergic and not the result of inadequate GAD staining.

Although all our injections labeled both GAD+ and GAD-negative IC cells, their proportions relative to one another and their distribution among the IC subdivisions varied according to the MG subdivision that was injected. The following sections describe the distributions of GAD+ and GAD-negative cells across the IC subdivisions following injections into each of the 4 MG subdivisions investigated.

### GAD-negative and GAD+ projections to individual MG subdivisions

**Figure 4:**
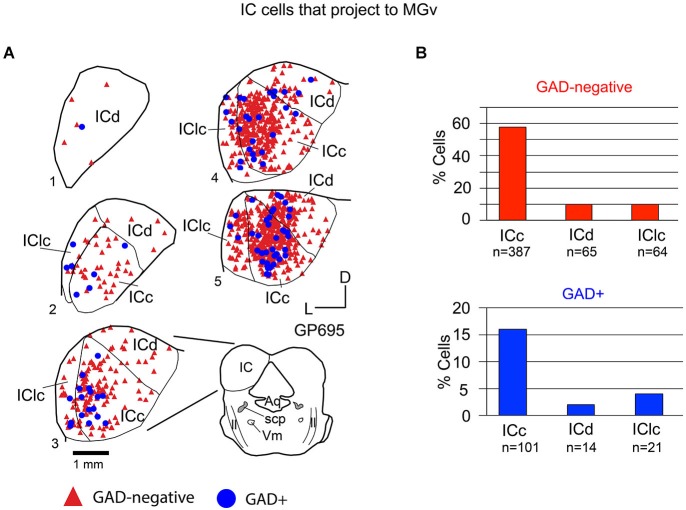
**(A)** Plots of transverse sections of the inferior colliculus (IC) illustrating the distribution of GAD+ (blue circles) and GAD-negative (red triangles) cells that were labeled by an injection of Red Beads into the ipsilateral ventral division of the medial geniculate body (MGv). Each symbol represents one retrogradely-labeled cell. Dorsal is up; 1 is the most caudal section; 5 is the most rostral section. Only the left IC is shown from each section except for section 3, which is accompanied by a full drawing of the brainstem cross section. Case GP695. **(B)** Histograms summarizing the distribution of GAD-negative and GAD+ IC cells that project to the MGv (data from GP695 and GP719; total *n* = 652 cells). Aq—aqueduct; ICc—IC central nucleus of the IC; ICd—IC dorsal cortex; IClc—lateral cortex of the IC; ll—lateral lemniscus; scp—superior cerebellar peduncle; V—motor nucleus of V.

Tracer injections restricted to the MGv labeled GAD-negative and GAD+ cells in each IC subdivision (Figure 4A). Overall, 79% of the tracer-labeled cells were GAD-negative. These GAD-negative cells were most numerous in the ICc, with the remaining cells split nearly evenly between the IClc and the ICd (Figure 4B, top graph). The GAD+ population constituted 21% of the retrogradely labeled cells. This population was also most prominent in the ICc with very few cells in the ICd and IClc (Figure 4B, bottom graph). Thus, both GAD-negative and GAD+ projections to the MGv originate primarily from the ICc.

**Figure 5:**
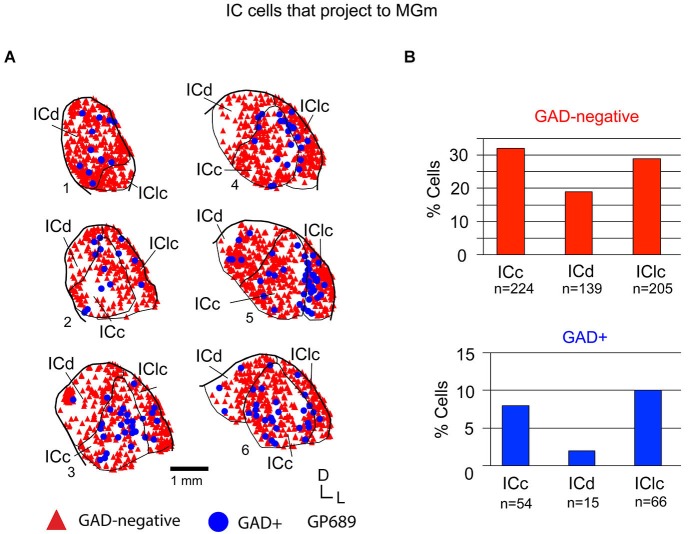
**(A)** Plots of transverse sections illustrating the distribution of GAD+ (blue circles) and GAD-negative (red triangles) inferior colliculus (IC) cells that were labeled by an injection of Green Beads into the ipsilateral MGm. Each symbol represents one retrogradely-labeled cell. Dorsal is up; 1 is the most caudal section; 6 is the most rostral section. Case GP689. **(B)** Histograms summarizing the distribution of GAD-negative and GAD+ IC cells that project to the MGm (data from GP689R; total *n* = 703 cells). ICc—IC central nucleus; ICd—IC dorsal cortex; IClc—lateral cortex of the IC.

Tracer injections restricted to the MGm labeled GAD-negative and GAD+ cells in each IC subdivision (Figure 5A). Overall, GAD-negative cells constituted 80% and GAD+ cells 20% of the tracer-labeled IC cells. As described above, the MGm was unique in receiving substantial inputs from two IC subdivisions (namely, the ICc and IClc). This pattern applied to both the GAD-negative and GAD+ populations of projecting cells (Figure 5B). The ICd contained the fewest cells in each population, with GAD+ cells particularly limited. Thus, the MGm receives substantial GAD+ and GAD-negative projections from both the ICc and the IClc, and a small, primarily GAD-negative projection from the ICd.

**Figure 6:**
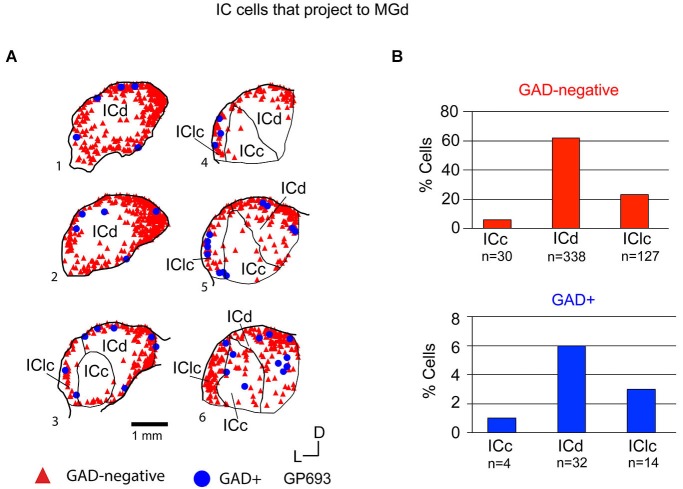
**(A)** Plots of transverse sections illustrating the distribution of GAD+ (blue circles) and GAD-negative (red triangles) inferior colliculus (IC) cells that were labeled by an injection of Red Beads into the ipsilateral MGd. Each symbol represents one retrogradely-labeled cell. Dorsal is up; 1 is the most caudal section; 6 is the most rostral section. Case GP693. **(B)** Histograms summarizing the distribution of GAD-negative and GAD+ IC cells that project to the MGd (data from GP693 and GP718; total *n* = 545 cells). ICc—IC central nucleus; ICd—IC dorsal cortex; IClc—lateral cortex of the IC.

Tracer injections restricted to the MGd labeled GAD-negative and GAD+ cells in each IC subdivision (Figure 6A). Overall, GAD-negative cells constituted 89% and GAD+ cells 11% of the tracer-labeled IC cells (Figure 6B). Both the GAD-negative and GAD+ populations were most prominent in the ICd (Figure 6B). Smaller subsets of both groups were located in the IClc, and the smallest proportions of each group were found in the ICc (Figure 6B). Thus, the MGd receives substantial GAD-negative and GAD+ projections primarily from the ICd and much less so from the IClc.

**Figure 7:**
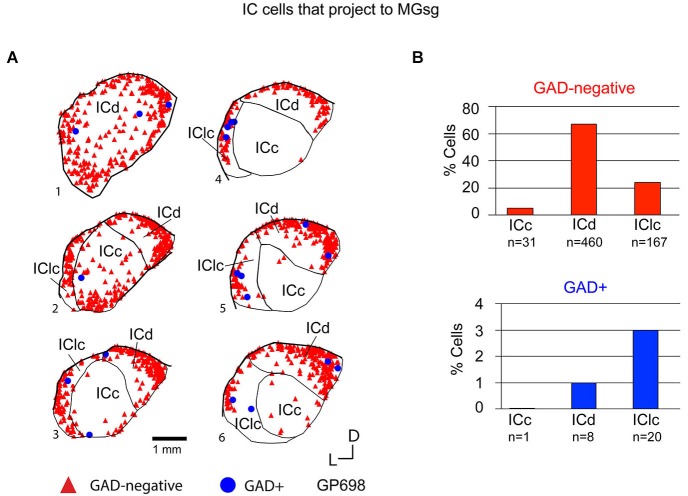
**(A)** Plots of transverse sections illustrating the distribution of GAD+ (blue circles) and GAD-negative (red triangles) inferior colliculus (IC) cells that were labeled by an injection of Red Beads into the ipsilateral MGsg. Each symbol represents one retrogradely-labeled cell. Dorsal is up; 1 is the most caudal section; 6 is the most rostral section. GP698. **(B)** Histograms summarizing the distribution of GAD-negative and GAD+ IC cells that project to the MGsg (data from GP696 and GP698; total *n* = 687 cells). ICc—IC central nucleus; ICd—IC dorsal cortex; IClc—lateral cortex of the IC.

**Figure 8:**
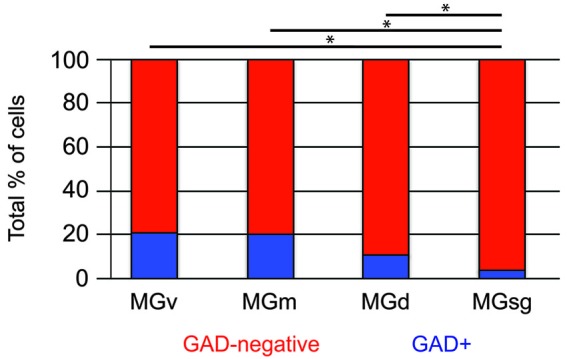
**Histogram showing the percentage of GAD+ cells in pathways from the IC to specific MG subdivisions**. Statistical comparisons were generated by chi-square independence tests along with *post hoc* pairwise chi-square tests to determine the likelihood that a cell in the IC that projected to the MGsg was significantly less likely to be GAD+ than if it were projecting to MGv, MGm or MGd. Statistical significance: *p < 0.001. MGd—dorsal division of the MG; MGm—medial division of the MG; MGsg—suprageniculate division of the MG; MGv—ventral division of the MG.

Tracer injections restricted to the MGsg labeled GAD-negative cells in each IC subdivision and GAD+ cells in the ICd and IClc (Figure 7A). Overall, GAD-negative cells constituted 96% and GAD+ cells 4% of the tracer-labeled cells. The GAD-negative cells were most numerous in the ICd, with most of the remainder in the IClc (Figure 7B, top graph). The rare GAD+ cells were located in the IClc and, less often, in the ICd (Figure 7B, bottom graph). Thus the MGsg is unique in receiving an extremely limited GABAergic projection from the IC. The prominent GAD-negative projection originates primarily from the ICd. We employed chi-square independence tests along with *post hoc* pairwise chi-square tests to determine the likelihood that a given IC cell projecting to the MGsg was GAD+. Results showed that a cell in the IC projecting to the MGsg was significantly less likely to be GAD+ than if it were projecting to MGv, MGm or MGd (Figure 8; *p* < 0.001).

## Discussion

Despite the common usage of guinea pigs in auditory research, there is little information about the organization of tectothalamic projections in this species. The current study examines the projections from specific IC subdivisions to 4 subdivisions of the MG in guinea pigs. Our first finding is that each MG subdivision receives input predominantly from one (or, for the MGm, two) IC subdivisions. These dominant connections closely reflect the parallel pathways described in several other species. Inputs from the non-dominant IC subdivisions represented 21–32% of the tectothalamic inputs to a particular MG subdivision. These inputs represent a possibility of cross-talk between the parallel pathways that could underlie functional integration. Our second objective focused on the presence of GAD-negative and GAD-positive tectothalamic cells. We asked whether these two subpopulations share similar patterns of projection to the MG. For all the MG subdivisions examined, the majority of IC inputs come from GAD-negative, presumptively glutamatergic, cells. GAD-positive, presumptive GABAergic, cells constituted 4–21% of the inputs to individual MG subdivisions. For projections to 3 of the MG subdivisions, the glutamatergic and GABAergic projections showed a similar distribution of inputs from the different IC subdivisions, differing only in the greater overall number of glutamatergic cells. For projections to the MGsg, GABAergic cells make only a minimal contribution, suggesting that the tectothalamic projections to this part of the MG is almost exclusively excitatory. In the following sections, we discuss technical aspects of our analysis, compare our findings to previous descriptions of the parallel tectothalamic pathways, and then consider some functional implications of these pathways and the differential contributions of GABAergic projections.

### Technical Considerations

Small volumes of tracer were used to ensure that deposits were located within a single MG subdivision. Such confined injections allow analysis without contamination by labeling cells that project to a neighboring subdivision. Of course, none of the restricted injections filled an MG subdivision entirely, so there is a risk that projections that terminate in only a portion of a subdivision could go undetected. The small injection volumes may also risk incomplete labeling because of limits to the sensitivity of tracers. We tried to minimize these limitations by using multiple tracers (green beads, red beads and FluoroGold). The beads are particularly valuable in this regard because of their high sensitivity and limited diffusibility in the tissue (Schofield, 2008). These characteristics allow relatively large amounts of tracer to be restricted to a small volume of tissue, resulting in many labeled cells. The fact that we obtained similar results across animals and across tracers, and that larger injections (involving multiple MG subdivisions) were consistent with the results from the small injections, suggests that our results are generally valid.

The GAD antibody used here has been validated in previous studies in guinea pigs (Xiong et al., 2008; Nakamoto et al., 2013; Mellott et al., 2014) and we believe that our tissue contained few false positive cells. Incomplete penetration of immunoreagents can lead to false negative staining, which could substantially affect quantitative analyses. We systematically limited our analysis such that, for each tissue section, labeled structures were analyzed only at tissue depths that included robust immunostaining. We conclude that GAD+ cells are GABAergic and that the GAD-negative cells are almost certainly non-GABAergic. Both anatomical and physiological data argue that the GAD-negative cells are glutamatergic. First, nearly all IC cells appear to be GABAergic or glutamatergic (Ito et al., 2011; Ito and Oliver, 2012). Second, stimulation of IC inputs to the MG can be blocked completely by pharmacological blockade of glutamate and GABA (Peruzzi et al., 1997). We conclude that most or all of the GAD-negative tectothalamic cells are glutamatergic.

### Parallel pathways in guinea pigs

The concept of parallel pathways in the upper auditory system is often traced to a seminal report by Calford and Aitkin (1983). These authors based their findings on the patterns of tectothalamic connections in cats, which they related to thalamocortical projections described in earlier studies. The concept thus encompasses pathways from midbrain to forebrain and has proven attractive and widely accepted (e.g., de Ribaupierre, 1997; Rouiller, 1997; Hu, 2003; Wenstrup, 2005). Extension of this concept to other species has often been based on thalamocortical (and corticothalamic) relationships (reviewed by, Rouiller, 1997), with relatively less information on the tectothalamic projections. Support for the existence of parallel pathways in guinea pigs comes from evidence for anatomical and physiological differences between the MG subdivisions and for differences in thalamocortical connections (Redies et al., 1989; Redies and Brandner, 1991; Edeline et al., 1999; He, 2001, 2003; Anderson et al., 2007). The present data indicate that tectothalamic projections in guinea pigs reflect the organization described in other species. The MGv and lemniscal pathway receive majority input from the ICc, the MGd/MGsg and the diffuse pathway get inputs mostly from the ICd, and the MGm and associated polysensory pathway get substantial inputs from both the IClc and the ICc. In all cases, smaller projections arise from the non-dominant IC subdivisions, providing potential opportunities for cross-talk between the parallel pathways (Figure 2).

### GABAergic and glutamatergic components of the parallel pathways

Our analysis of glutamatergic vs. GABAergic components of the tectothalamic pathway lead to a few additional conclusions. First, the glutamatergic projections are numerically dominant and, not surprisingly, closely match the overall projection patterns (i.e., the subdivision-specific connections). The GABAergic projections are smaller, comprising 4–21% of the projections to a given MG subdivision. These projections largely reflect the overall parallel pathways, with the notable difference that very few GABAergic cells project to the MGsg (this difference is one basis for distinguishing the MGsg from the MGd and the other MG subdivisions; this is discussed in more detail below). Except for the MGsg, each MG subdivision receives both glutamatergic and GABAergic projections from the same subset of IC subdivisions. What function(s) are served by this convergence of excitation and inhibition?

Inhibitory inputs to the MG, like those to other regions of the auditory system, are considered critical for temporal processing of acoustic signals (Venkataraman and Bartlett, 2013). *In vitro* studies have demonstrated that ascending excitatory and inhibitory projections (presumed tectothalamic inputs) converge on cells in multiple MG subdivisions (Bartlett and Smith, 1999, 2002; Smith et al., 2007; Venkataraman and Bartlett, 2013). In general, excitation or inhibition can arrive first, suggesting that the inhibitory inputs could influence both the onset and sustained portions of neuronal responses to sound. Moreover, different patterns of convergence occur, with some cells dominated by excitation, others dominated by inhibition (without any sign of ascending excitation) and the remaining cells showing more evenly mixed interactions. Temporal processing could be expected to play critical roles in all the parallel auditory pathways (Lennartz and Weinberger, 1992; Abrams et al., 2011), and in fact inhibitory/excitatory convergence has been seen in all MG subdivisions. However, the MG subdivisions differ in the relative numbers of cells that show the different patterns of excitatory and inhibitory interaction (discussed in Smith et al., 2007). The present results show differences in the excitatory and inhibitory projections from specific IC subdivisions to four large MG subdivisions, supporting the concept of parallel pathways and the conclusion that the MG subdivisions serve distinct functions.

### Crosstalk between parallel pathways

The presence of non-dominant projections, i.e., small projections that connect IC and MG subdivisions less heavily than the dominant projections, have been noted since the earliest descriptions of parallel pathways (e.g., Calford and Aitkin, 1983). Such cross-talk could allow for integration of information carried in different pathways, or allow activity in one pathway to influence processing in another pathway. Our results from GAD staining show that both GABAergic cells and glutamatergic cells contribute to the non-dominant connections described above. Thus, crosstalk between the pathways could include both excitatory and inhibitory components. A common role of inhibitory projections in many brain areas is lateral inhibition, and one might predict that the GABAergic projections serve to heighten the contrast between various channels and promote transmission through a particular channel. Both GABAergic and glutamatergic projections could allow for integration of information carried in the different channels. Such speculations await further insights into the role of the GABAergic and glutamatergic projections within channels as well as through crosstalk projections.

As mentioned above, the near absence of GABAergic tectothalamic projections distinguishes the MGsg from the other MG subdivisions. Previous studies have distinguished the MGsg based on connections with other regions of the brainstem, especially regarding strong projections to the MGsg from the superior colliculus (Tanaka et al., 1985; Hicks et al., 1986; Hoshino et al., 2010) and the sagulum (Morest, 1965). Examination of these regions in our experiments suggests that the MGsg in guinea pigs receives similar inputs. These connections suggest that the MGsg may play a role in integrating auditory and visual information and contribute to orientation or attention. The present results suggest that auditory tectothalamic contributions to these functions are carried out mainly by excitatory projections.

In summary, the present data suggest that tectothalamic projections in guinea pigs can be conceptualized by the same parallel pathways described in other species. Both excitatory and inhibitory projections contribute to these pathways and may provide a basis for more refined definitions and more complete understanding of the interactions of these pathways in the thalamus. The concept of parallel pathways has proven valuable for understanding many aspects of sensory processing. An interesting question for future work will be to determine the extent to which the current concept of parallel auditory pathways can accommodate new data on both subcortical and cortical connections of the MG.

## Author contributions

Designed research, wrote the paper: Jeffrey G. Mellott, Brett R. Schofield; performed research, analyzed data: all authors.

## Conflict of interest statement

The authors declare that the research was conducted in the absence of any commercial or financial relationships that could be construed as a potential conflict of interest.

## Abbreviations

AQ: cerebral aqueduct
FB: Fast Blue
FG: FluoroGold
GABA: gamma-aminobutyric acid
GAD: glutamic acid decarboxylase
GB: Green Beads
IC: inferior colliculus
ICc: central nucleus of the inferior colliculus
ICd: dorsal cortex of the inferior colliculus
IClc: lateral cortex of the inferior colliculus
ll: lateral lemniscus
MG: medial geniculate body
MGd: dorsal division of medial geniculate
MGm: medial division of medial geniculate
MGsg: suprageniculate division of medial geniculate
MGv: ventral division of medial geniculate
RB: Red Beads
scp: superior cerebellar peduncle
Vm: motor trigeminal nucleus.
